# Vitrectomy with internal limiting membrane peeling vs no peeling for Macular Hole-induced Retinal Detachment (MHRD): a meta-analysis

**DOI:** 10.1186/s12886-015-0048-5

**Published:** 2015-06-20

**Authors:** Jing Su, Xinquan Liu, Lijun Zheng, Hongping Cui

**Affiliations:** Department of Ophthalmology, Longhua Hospital, Shanghai University of Traditional Chinese Medicine, No. 375 South Wanping Road, Shanghai, 200032 P.R. China; Department of General Surgery, Shanghai Tenth people’s Hospital, Tongji University, No. 301 Middle Yanchang road, Shanghai, 200072 P.R. China; Department of Ophthalmology, Shanghai East Hospital, Tongji University, No.150 Jimo Road, Shanghai, 200120 P.R. China

**Keywords:** Macular hole-induced retinal detachment, Vitrectomy, Internal limiting membrane peeling, Meta-analysis

## Abstract

**Background:**

we conducted our meta-analysis of published studies to assess existing evidence about the efficacy and safety of vitrectomy with ILM peeling vs. that of vitrectomy with no ILM peeling for Macular hole-induced retinal detachment.

**Methods:**

Databases, including Pubmed, Cochrane Library, Ovid, Web of Science, Wanfang and CNKI, were searched to identify studies comparing outcomes following vitrectomy with ILM peeling and that with no ILM peeling for macular hole-induced retinal detachment. The meta-analysis was performed by RevMan 5.1.

**Results:**

Six comparative studies comprising 180 eyes were identified. It was indicated that the rate of retinal reattachment (Odds ratio (OR) = 3.03, 95 % Confidence interval (CI):1.35 to 6.78; *P* = 0.007) and macular hole closure (OR = 6.74, 95 % CI:3.26 to 13.93; *P* < 0.001) after initial surgery was higher and the rate of recurrent retinal detachment (OR = 0.08, 95 % CI:0.02 to 0.30; *P* = 0.0002) was lower in the group of vitrectomy with ILM peeling than that in the group of vitrectomy with no ILM peeling. However, the improved BCVA (Weighted mean difference (WMD) = 0.14, 95 % CI: −0.20 to 0.47; *P* = 0.42) and the rate of postoperative complications were similar between the two groups.

**Conclusion:**

Vitrectomy with internal limiting membrane peeling is an efficient and safe procedure for macular hole-induced retinal detachment.

## Background

Macular hole-induced retinal detachment (MHRD), also named retinal detachments resulting from macular hole is usually a vision-threatening complication to highly myopic eyes, which is more common in Asian adult population [1]. In the past years, MHRD was presumed as a rare disease according to the paucity of literatures. But with the OCT routinely used to evaluate RDs preoperatively, macular holes were found more frequently in RD [2]. The important causative factors of MHRD might be related to the tangential traction caused by a premacular membrane or fibrosis and the inverse traction caused by the posterior staphyloma [3–5]. More recent analysis by OCT showed that tangential traction of the vitreous cortex behind the vitreous pocket contributed to the development of MHRD [3–6]. In highly myopic eyes, the elongation of the axial length of the eye and the development of a posterior staphyloma result in thinning of the retina and choroid, which also leads to the development of MHRD [3, 7].

Since the early 1990s, pars plana vitrectomy, gas endotamponade, and epiretinal membrane removal had been used in retinal detachment related to a macular hole [8–13]. However, the primary success rate of retinal reattachment (43.9–75 %) was not as high as expected and the visual outcomes in some cases were poor [11–15]. To facilitate macular hole closure, removal of the internal limiting membrane (ILM) had been used a surgical adjunct primarily to counter surface traction and promote the closure of the macular hole in the past fifteen years [16–18]. It was proposed that removing the internal limiting membrane (ILM) ensures the complete removal of any overlying ERM adjacent to an indiopathic macular hole and the vitreous traction on the retina [19–24].

Although an increasing number of PPV with ILM peeling has been reported treating retinal detachments resulting from a macular hole with better retinal reattachment and visual acuity [25–29], ILM peeling has been shown to lead to small but noticeable anatomic and functional changes in the peeled area of the retina, which should also be considered in the risk–benefit analysis. There is still debate among vitreoretinal surgeons about whether and when to peel the ILM in MHRD cases. The removal of the ILM may increase the incidence of postoperative complications, including the development of a MH and a MHRD [21]. Moreover, some functional outcomes such as postoperative scotomas and dissociated optic nerve fibre layer (DONFL) should also be considered in the risk–benefit analysis [30, 31]. Most studies have been limited by small sample size and a single institution design, so consensus has not been reached as to the necessity of ILM peeling in MHRD. To overcome these limitations, we conducted this meta-analysis of published studies to assess existing evidence about the efficacy and safety of vitrectomy with ILM peeling vs that of vitrectomy with no ILM peeling for macular hole-induced retinal detachment.

## Methods

### Literature search

A literature search was performed to identify all relevant prospective or retrospective studies that compared outcomes following vitrectomy with ILM peeling and that with no ILM peeling for macular hole-induced retinal detachment. The Pubmed, Cochrane Library, Ovid, Web of Science, Wanfang and CNKI databases were searched systematically for all articles published before June 2014. The following terms were used for the search: “retinal detachment”, “macular hole”, “myopic”, “macular hole-induced retinal detachment”, “internal limiting membrane peeling” and “vitrectomy”. Only studies in the English or Chinese language were considered for inclusion. Reference lists of all retrieved articles were manually searched to broaden the search. All abstracts, studies and citations scanned were reviewed.

### Data extraction and assessment of study quality

Two reviewers independently extracted the data from each study. The following information was extracted from each study: first author, year of publication, study design, inclusion and exclusion criteria, quality of study, study population characteristics, number of subjects in vitrectomy with ILM peeling group and vitrectomy with no ILM peeling group, baseline characteristics of the patients such as duration of symptoms, refractive error, axial length and preoperative BCVA in each group, postoperative data. Discrepancies between the two reviewers were resolved by discussion and consensus with the corresponding author.

Since most of our selected studies were non-randomized surgical research, the quality of each included trial was accessed using methodological index for non-randomized studies (MINORS) [32]. This validated index involves 12 items, the first eight items specifically designed for non-comparative studies and the remaining four items applied to comparative studies. Items are scored as 0 (not reported), 1 (reported but inadequate) and 2 (reported and adequate). The maximum ideal score for comparative studies is 24. It is important to appreciate that such scoring system was use in the quality comparison of nonrandomized research because other quality grading according to levels of evidence does not provide adequate stratification [32]. We evaluated each study with a quality score and the score of 12 or more indicated a higher quality study.

### Criteria for inclusion and exclusion

To be included in this meta-analysis, studies had to fulfill the following criteria: (1) compare outcomes of patients receiving vitrectomy with ILM peeling with those of patients receiving vitrectomy with no ILM peeling for macular hole-induced retinal detachment; (2) report on at least one of the outcome measures mentioned below; and (3) if multiple studies were reported by the same institution and/or authors, either the best quality or the most recent publication was included in our analysis.

Noncomparative studies were excluded. Abstracts, letters, editorials and experts opinions and reviews without original data were excluded. The following studies or data were also excluded: (1) studies included cases with both MHs and peripheral breaks; (2) the outcomes and parameters of patients were not clearly reported; (3) significant differences existed in duration of symptoms, refractive error, axial length and preoperative BCVA between vitrectomy with ILM peeling group and vitrectomy with no ILM peeling group; (4) If end points were not comparable, (5) if it was impossible to extract or calculate appropriate data from the published results.

### Outcomes of interest

The following outcomes were used to compare between the group of vitrectomy with ILM peeling and that of vitrectomy with no ILM peeling. (1) data of efficacy, including rate of retinal reattachment after initial surgery, rate of macular hole closure after initial surgery, improved BCVA and rate of recurrent retinal detachment; (2) data of safety, the rate of postoperative complication such as retinal breaks, ERM (epiretinal membrane), cataract and intraocular pressure rise.

### Statistical analysis

We used the Cochrane Collaboration’s Review Manager Software (RevMan Version 5.1) for the data analysis. Dichotomous variables were analyzed by using estimation of odds ratios with a 95 % confidence interval (95 % CIs) and continuous variables using weighted mean difference (WMD) with 95 % CIs. For studies that presented continuous data such as median and range values, we converted these data to the mean and standard deviation by using the method of Hozo et al. [33]. Thus all continuous data were standardized for analysis.

The homogeneous test of effects was performed using *χ*^2^ test, with *P* < 0.05 and I^2^ > 50 % indicating significant heterogeneity. A fixed-effects model was used when no heterogeneity was detected, which meant that there was no variances among studies. If any heterogeneity existed, a random-effects model, which leads to wider CIs than the fixed-effects model, was used for the meta-analysis. Presence of publication bias was evaluated qualitatively by a funnel plot. We also systematically describe and assess the results that are not appropriate to be combined in the meta-analysis.

## Results

### Selection of studies

**Fig. 1:**
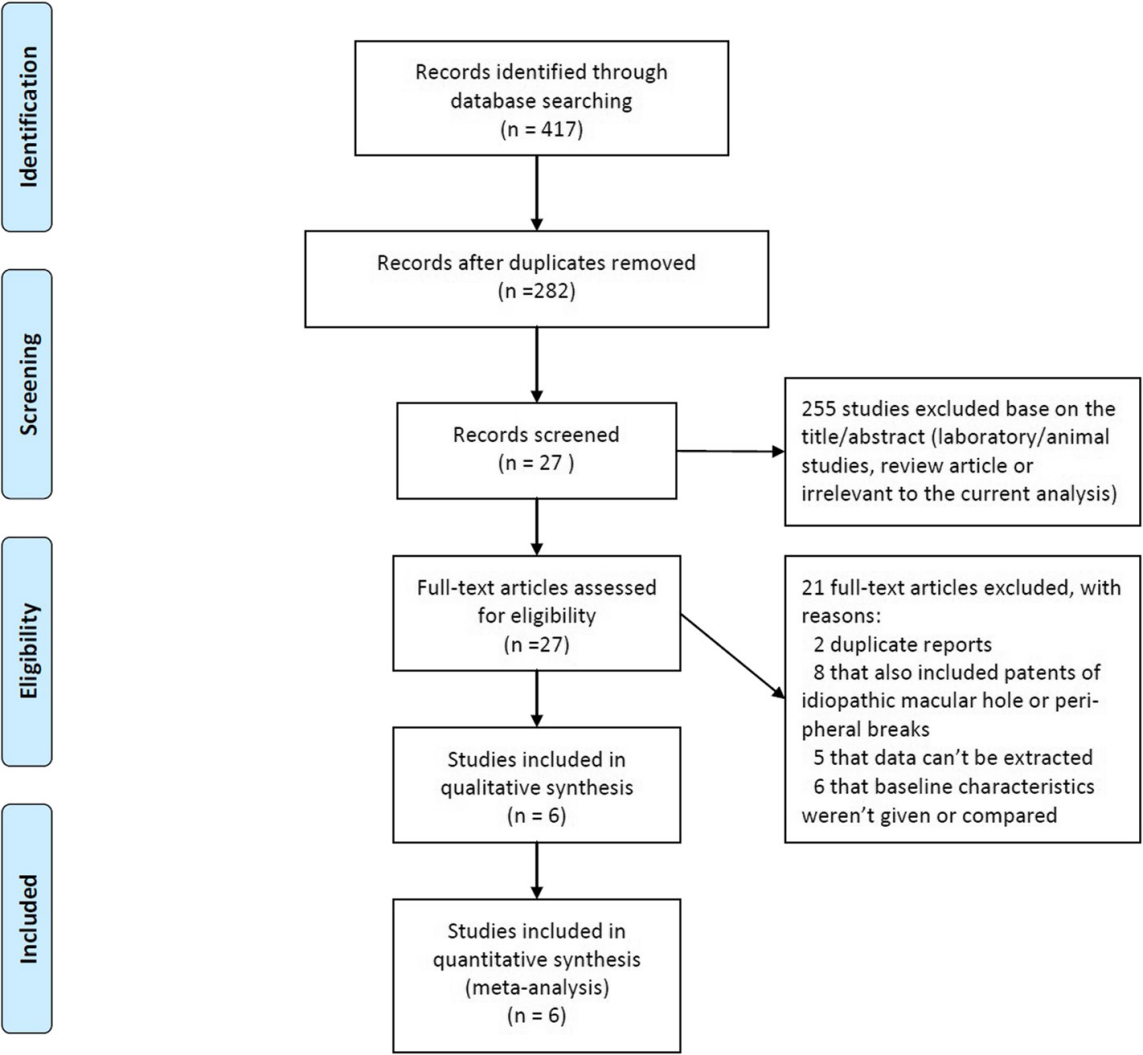
Flow diagram of the trial selection process

The initial search yielded 417 relevant studies. But most of these studies were not suitable for our analysis because they included duplicates, lab or animal studies, case reports, review and other study subjects irrelevant to our title. After screening all titles, abstracts and full-test, 411 publications were excluded according to the selection criteria and a total of 6 studies [28, 34–38] were retrieved for more detailed evaluation. The search process is illustrated in Fig. 1.

### Characteristics and baseline of the included studies

In total 6 studies [28, 34–38], 180 eyes (92 eyes with ILM peeling, 88 eyes with no ILM peeling) were included with retinal detachment resulting from macular hole. The characteristics of these 6 studies are summarized in Table 1. None of the studies were randomized controlled trials. Five studies [28, 35–38] were conducted in China, one in Japan [34]. The sample size of each study varied from 11 to 52. All the studies included in the meta-analysis were considerably well conducted and had balanced populations.

**Table 1:** Characteristics of studies included in this meta-analysis (VT = vitrectomy, ILMP = internal limiting membrane peeling)

Study	Year	Country	Study type	Group	No. of eyes	Mean age (yr)	Duration of symptoms (days)	Refractive error (diopters)	Axial length (mm)	LogMAR BCVA	Tamponade agents	Dye for ILM stained	Duration of follow-up (months)
Uemoto et al. [34]	2004	Japan	Retro	VT with ILMP	13	63.5	90	−11.1	29.1	1.58	SF6/C3F8	ICG	66.6
				VT without ILMP	12	56.8	135	−15.3	29.7	1.4			23.9
Liu et al. [35]	2009	China	Retro	VT with ILMP	12	59.0	NR	−10.0	29.9	1.53	SF6/C3F8	ICG	NR
				VT without ILMP	13	58.0	NR	−10.5	30.6	1.47			NR
Li et al. [28]	2010	China	Retro	VT with ILMP	10	64.4	NR	−11.25	28.3	1.70	C3F8	trypan blue	15.5
				VT without ILMP	9	62.0	NR	−16.65	29.6	1.74			43.0
Yu et al. [36]	2010	China	Retro	VT with ILMP	26	56.2	94	−13.2	28.3	1.72	C3F8	ICG	20.7
				VT without ILMP	26	52.8	101	−13.0	28.2	1.69			22.5
Fan et al. [37]	2011	China	Retro	VT with ILMP	6	NR	NR	−11.7	28.7	NR	C3F8	NS	NR
				VT without ILMP	5	NR	NR	−16.0	26.9	NR			NR
Wei et al. [38]	2013	China	Retro	VT with ILMP	25	57.3	51	−12.9	NR	1.90	C3F8	ICG	NR
				VT without ILMP	23	55.7	51	−12.1	NR	1.80			NR

The baseline characteristics of each included trial, such as duration of symptoms, refractive error, axial length and preoperative BCVA were found to be equivalent between the group of vitrectomy with ILM peeling and the group of vitrectomy with no ILM peeling. Meanwhile, analysis of the pooled data revealed that the two groups did not differ significantly and there was no statistical heterogeneity between the studies (Table 2).

**Table 2:** Analysis of baseline characteristics between the group of vitrectomy with ILM peeling and the group of vitrectomy with no ILM peeling

Baseline characteristics	No. of studies	No. of eyes	Study heterogeneity		Analysis model	Analysis of the pooled data	
			I^2^, %	P value		WMD (95 % CI)	P value
Duration of symptoms	3	125	0	0.69	fixed-effects	−0.05 (−0.50, 0.39)	0.82
Refractive error	6	180	46	0.10	fixed-effects	0.85 (−0.13, 1.83)	0.09
Axial length	5	132	18	0.30	fixed-effects	−0.34 (−1.04, 0.37)	0.35
Preoperative BCVA	4	150	0	0.88	fixed-effects	0.08 (−0.04, 0.20)	0.19

### Quality assessment

The methodologic quality of the included trials is explained comprehensively in Table 3. In general, the quality of the studies was moderate to good (all >12). All data were analyzed in accordance with intention-to-treat principle.

**Table 3:** MINORS for assessing quality of included studies

Methodological item for non-randomized studies	Uemoto et al. [34]	Liu et al. [35]	Li et al. [28]	Yu et al. [36]	Fan et al. [37]	Wei et al. [38]
1. A clearly stated aim	2	2	2	2	2	2
2. Inclusion of consecutive patients	2	2	2	2	2	2
3. Prospective collection of data	0	0	0	0	0	0
4. Endpoints appropriate to the aim of the study	2	2	2	2	2	2
5. Unbiased assessment of the study endpoint	0	0	0	0	0	0
6. Follow-up period appropriate to the aim of the study	2	0	2	2	0	0
7. Loss to follow up less than 5 %	2	0	0	2	0	1
8. Prospective calculation of the study size	0	0	1	0	0	1
9. An adequate control group	2	2	2	2	2	2
10. Contemporary groups	2	2	2	2	2	2
11. Baseline equivalence of groups	2	2	2	2	1	2
12. Adequate statistical analyses	2	2	2	2	2	2
Total score	18	14	17	18	13	16

### Meta-analysis of efficacy outcomes

**Fig. 2:**
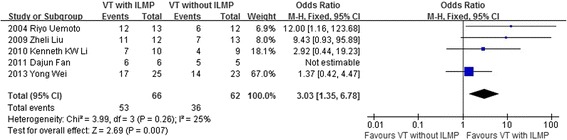
A forest plot showing the rate of retinal reattachment after initial surgery between the group of vitrectomy with ILM peeling and the group of vitrectomy with no ILM peeling for retinal detachments resulting from a macular hole. VT = vitrectomy, ILMP = internal limiting membrane peeling

The pooled data from 5 studies including 128 eyes in the meta-analysis indicated that the group of vitrectomy with ILM peeling had higher rate of retinal reattachment after initial surgery than the group of vitrectomy with no ILM peeling (OR = 3.03, 95 % CI: 1.35 to 6.78; *P* = 0.007) and there was no statistical heterogeneity between the two groups (heterogeneity *P* = 0.26, I^2^ = 25 %) (Fig. 2).

**Fig. 3:**
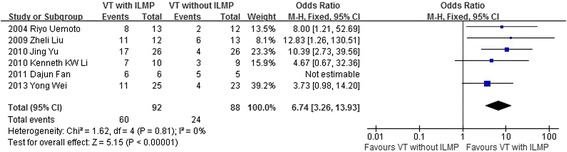
A forest plot showing the rate of macular hole closure after initial surgery between the group of vitrectomy with ILM peeling and the group of vitrectomy with no ILM peeling for retinal detachments resulting from a macular hole. VT = vitrectomy, ILMP = internal limiting membrane peeling

The rate of macular hole closure after initial surgery was reported in 6 studies including 180 eyes. There was no statistical heterogeneity between the studies (heterogeneity *P* = 0.81, I^2^ = 0 %). By using a fixed effects model, it was indicated that the rate of macular hole closure after initial surgery was higher in the group of vitrectomy with ILM peeling than that in the group of vitrectomy with no ILM peeling (OR = 6.74, 95 % CI: 3.26 to 13.93; *P* < 0.001) (Fig. 3).

**Fig. 4:**
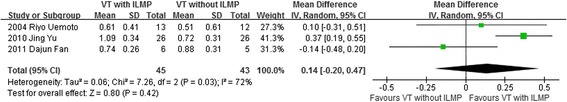
A forest plot showing the improvement of BCVA after surgery between the group of vitrectomy with ILM peeling and the group of vitrectomy with no ILM peeling for retinal detachments resulting from a macular hole. VT = vitrectomy, ILMP = internal limiting membrane peeling

In 3 studies, it was indicated that there was no significant difference in the improvement of BCVA after surgery between the two groups. Analysis of the extracted data revealed that there was statistical heterogeneity between the studies (heterogeneity *P* = 0.03, I^2^ = 72 %), which may have resulted from variations in the method used to measure the visual acuity and the data conversion from other unit to logMAR. Patients undergoing vitrectomy with ILM peeling experience a similar improvement of BCVA of those undergoing vitrectomy with no ILM peeling. The two groups did not differ significantly in the regard (WMD = 0.14, 95 % CI: −0.20 to 0.47; *P* = 0.42) using the random effects model (Fig. 4).

**Fig. 5:**
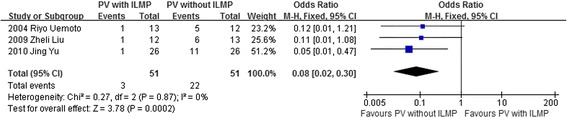
A forest plot showing the rate of recurrent retinal detachment after initial surgery between the group of vitrectomy with ILM peeling and the group of vitrectomy with no ILM peeling for retinal detachments resulting from a macular hole. VT = vitrectomy, ILMP = internal limiting membrane peeling

The pooled data from 3 studies including 102 eyes in the meta-analysis indicated that the group of vitrectomy with ILM peeling had lower rate of recurrent retinal detachment after initial surgery than the group of vitrectomy with no ILM peeling (OR = 0.08, 95 % CI: 0.02 to 0.30; *P* = 0.0002) and there was no statistical heterogeneity between the two groups (heterogeneity *P* = 0.87, I^2^ = 0 %) (Fig. 5).

### Meta-analysis of safety outcomes

Five studies reported postoperative complications such as retinal breaks, ERM (epiretinal membrane), cataract and intraocular pressure rise. As is shown in Table 4, we analyzed the pooled data of complications respectively and revealed that the two groups did not differ significantly in the regard of using the fixed effects model. It is important to note that heterogeneity testing indicated no significant heterogeneity between the two groups.

**Table 4:** Comparison of safety outcomes between the group of vitrectomy with ILM peeling and the group of vitrectomy with no ILM peeling

Safety outcomes	No. of studies	No. of eyes	Study heterogeneity		Analysis model	Analysis of the pooled data	
			I^2^, %	P value		OR (95 % CI)	P value
Retinal breaks	2	73	0	1.00	fixed-effects	0.91 (0.17 to 5.02)	0.92
Epiretinal membrane	2	73	0	0.96	fixed-effects	0.31 (0.07 to 1.44)	0.13
Postoperative cataract	2	100	42	0.19	fixed-effects	1.10 (0.41 to 2.90)	0.85
Intraocular pressure rise	2	62	0	0.33	fixed-effects	0.89 (0.28 to 2.79)	0.84

### Testing for publication bias

**Fig. 6:**
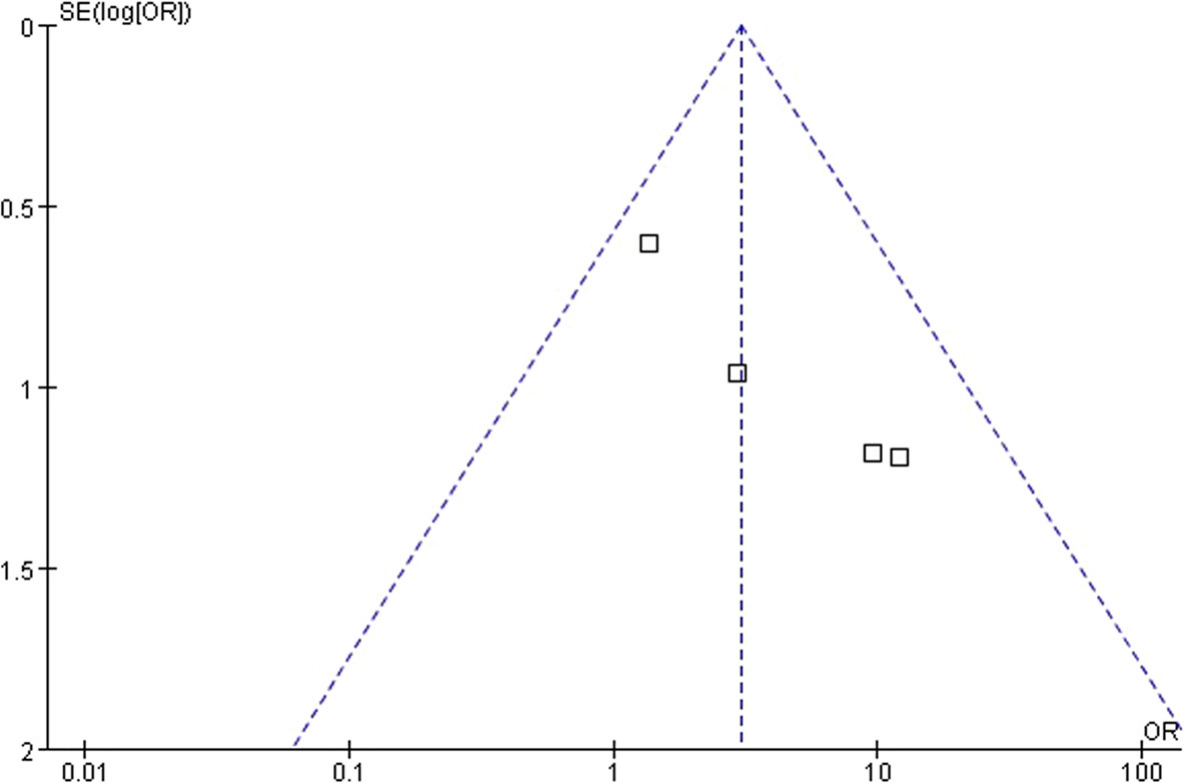
A funnel plot for the result from the studies comparing the rate of retinal reattachment showing no significant publication bias. SE = standard error, OR = odds ratio

A funnel plot of the macular hole closure rate in including studies demonstrated symmetry, which indicated no serious publication bias (Fig. 6).

## Discussion

Gonvers and Machemer [39] first introduced the surgical procedures for treating retinal detachments (RDs) resulting from a macular hole. Since then, pars plana vitrectomy, gas endotamponade, and epiretinal membrane removal has been widely accepted as the treatment for retinal detachment related to a macular hole [8–13]. Despite the universality of pars planavitrectomy for MHRD, the primary success rate of retinal reattachment was not as high as expected. Previous studies on retinal detachment related to high myopia in patients with macular hole have demonstrated that vitreous surgery can lead to anatomic macular hole closure, with the primary anatomic closure rate ranging from 46–75 % [8–15]. Kadonosono et al. first reported ILM peeling with the assistance of indocyanine green and sulfur hexafluoride gas injection for retinal detachment related to high myopia in patients with macular hole with a high reattachment success rate of 91 % [25]. However most studies have been limited by small sample size and a single institution design, consensus has not been reached as to the necessity of ILM peeling in MHRD.

Since macular hole-induced retinal detachment is relatively uncommon, it is unlikely to perform large scale studies or randomized studies to study on the necessity of ILM peeling in it. So we design our meta-analysis to determine the efficacy and safety of ILM peeling in macular hole-induced retinal detachment. In this meta-analysis, we pooled data from 7 studies and examined eight factors associated efficacy and safety: rate of retinal reattachment after initial surgery, rate of macular hole closure after initial surgery, improved BCVA, rate of recurrent retinal detachment and rate of postoperative complication such as retinal breaks, ERM (epiretinal membrane), cataract and intraocular pressure rise.

We realized that the efficacy and safety outcomes were associated with the baseline characteristics of eyes including in studies such as duration of symptoms, refractive error, axial length and preoperative BCVA. So we excluded the studies which existed significant difference in duration of symptoms, refractive error, axial length and preoperative BCVA between the group of vitrectomy with ILM peeling and that of vitrectomy with no ILM peeling. Before we analyzed the outcomes of efficacy and safety, we compared the duration of symptoms, refractive error, axial length and preoperative BCVA between the two groups and found no significant difference. According to this result we consider that the groups have comparability and the conclusion below was reasonable.

Our meta-analysis summarized the efficacy and safety outcomes of ILM peeling in vitrectomy with a total of 113 case eyes and 102 control eyes. The result indicated that the rate of retinal reattachment and macular hole closure after initial surgery was higher and the rate of recurrent retinal detachment was lower in the group of vitrectomy with ILM peeling than that in the group of vitrectomy with no ILM peeling (Figs. 2–3, 5). However, the improved BCVA and the rate of postoperative complications were similar between the two groups (Fig. 4, Table 4).

Macular hole-induced retinal detachment (MHRD) is usually a vision-threatening complication to highly myopic eyes, which is more common in Asian adult population [1]. The important causative factors of MHRD might be related to anterior-posterior traction of the vitreous on the macular area of the retina or fibrosis and the inverse traction caused by the posterior staphyloma [3–5]. Histologic studies of excised posterior vitreous cortex in the eyes with MHRD have shown that the fibrous astrocytes made up the majority of cells, and the cortical vitreous contained abundant newly formed collagen including fibrous long-spacing collagen surrounded by sparsely distribute native vitreous collagen [40]. These findings indicated that the removal of the vitrous cortex should reduce the tangential traction and resolve the myopic traction maculopathy. In highly myopic eyes, the elongation of the axial length of the eye and the development of a posterior staphyloma result in thinning of the retina and choroid, which then leads to the development of MHRD [3, 7]. It was found that the posterior vitreous cortex or a thin ERM was adherent to the detached retina during surgery in all cases [34]. Thus, ILM peeling is considered to ensure the complete removal of the overlying residual vitreous cortex or ERMs and to relieve the tangential traction of residual prefoveal vitreous after posterior vitreous detachment of the contraction of epiretinal cellular constituents adjacent to the macular hole, resulting in closing the macular hole and aiding in the recovery of macular shape [41]. However, it is usually difficult to remove a thin and fragile ERM or posterior vitreous cortex completely from a detached retina. It was supposed that without ILM peeling, the remaining vitreous may act as a scaffold for the epiretinal membrane, thereby exerting traction on both the MH and retina in the posterior pole, thus limiting MH closure or even promoting reopening.

In our meta-analysis, the improved BCVA was not significantly difference between the group of vitrectomy with ILM peeling and the group of vitrectomy with no ILM peeling (Fig. 4). No visual acuity improvement difference are likely explained by the fact that patients whose macular hole had not closed or who developed recurrent retinal detachment after initial surgery in the group of no ILM peeling were ethically allowed to receive second surgery including ILM peeling in clinical study. Thus the outcome of improved BCVA was observed similar in the last data point of follow-up between the two groups [42]. The ILM is the basal lamina of the Müller cells, and the Müller cell cone, which is an inverted cone-shaped zone of specialized Müller cells that form the base of the fovea [43], serves as a plug that binds the photoreceptor cells together in the macula and supports the macula structurally [44]. ILM peeling may decrease the structural support of the macula [21], reduce the amplitude of the local electroretinogram (ERG) [45] and dissociate optic nerve fiber layer [30]. Despite the anatomic change, there are no functional consequences have been attributed to these findings. However, we supposed that these anatomic changes had potential negative effect on the improvement of BCVA in the group of vitrectomy with ILM peeling.

In our meta-analysis, the rate of postoperative complications was not significantly different between the group of vitrectomy with ILM peeling and the group of vitrectomy with no ILM peeling (Table 4). We supposed that the surgeons’ experience increasing and the dye application in ILM peeling made the adverse effect of ILM peeling be avoided. Retinal breaks, ERM (epiretinal membrane), cataract and intraocular pressure rise were the most common complications after the progresses. However, all the major surgical complications were few both in the two groups.

The results of the present meta-analysis should be interpreted with caution because of several limitations. First, all the studies available for this meta-analysis were retrospective studies, so there was a possibility of evident selection bias and observer bias with regard to the adoption of the operative approach. The surgeons might deal the eyes which had larger macular hole size, higher refractive errors and longer symptom duration with no ILM peeling to avoid postoperative application such as retinal breaks and cataract. Second, as is known, successful vitrectomy with or without ILM peeling depends on individual experiences. Surgeons with varying expertise from different clinical centers were included our study. Therefore, the efficacy outcomes such as rate of retinal reattachment after initial surgery, rate of macular hole closure after initial surgery, improved BCVA and rate of recurrent retinal detachment might be affected. The problem of intersurgeon variability, which most of the clinical trials might encounter was difficult to solve. Third, although our funnel plot showed that publication bias is unlikely, it is important to bear in mind that publication bias usually existed in meta-analysis based on published studies. Finally, converting non-normally distributed statistics (median and range) to normally distributed statistics (mean and SD) may be a cause of bias in our analysis.

## Conclusions

In conclusion, the present meta-analysis of published studies has shown that vitrectomy with internal limiting membrane peeling is an efficient and safe procedure for the treatment of macular hole-induced retinal detachment with high rate of retinal reattachment and macular hole closure, lower rate of recurrent retinal detachment as compared to the procedure of vitrectomy with no internal limiting membrane peeling. Therefore, vitrectomy with ILM peeling may be a preferred treatment for macular hole-induced retinal detachment.
